# Feeding of high-quality hay modulates hepatic lipid and energy metabolism in weaned dairy calves

**DOI:** 10.3168/jdsc.2025-0793

**Published:** 2025-07-16

**Authors:** Barbara U. Metzler-Zebeli, Arife Sener-Aydemir, Georg Terler, Qendrim Zebeli

**Affiliations:** 1 Centre for Veterinary Systems Transformation and Sustainability, Clinical Department for Farm Animals and Food System Science, University of Veterinary Medicine Vienna, 1210 Vienna, Austria; 2 Centre for Animal Nutrition and Welfare, Clinical Department for Farm Animals and Food System Science, University of Veterinary Medicine Vienna, 1210 Vienna, Austria; 3 Institute for Livestock Research, Agricultural Research and Education Centre Raumberg-Gumpenstein, 8952 Irdning-Donnersbachtal, Austria

## Abstract

The present study aimed to investigate the effects of feeding different hay qualities with or without concentrate supplementation on the mRNA expression of genes related to hepatic lipid and glucose metabolism and cellular energy status in weaned calves. Holstein Friesian calves (5 per dietary group) were fed 4 solid diets: (1) 100% medium-quality hay (MQH; 9.4 MJ of ME, 149 g CP, 522 g NDF/kg of DM); (2) 100% high-quality hay (HQH; 11.2 MJ of ME, 210 g CP, 455 g NDF/kg of DM); (3) 30% medium-quality hay + 70% starter concentrate (MQHC); and (4) 30% high-quality hay + 70% starter concentrate (HQHC). Calves were slaughtered at 100 ± 4 d of age and an average BW of 128.8 ± 19.8 kg. Liver tissue was collected, snap-frozen in liquid N_2_, and used in the mRNA expression experiment. The trend for lower mRNA expression of AMP-kinase with HQH compared with MQH indicated higher energetic status of hepatocytes with HQH, which probably triggered the increased mRNA expression of enzymes responsible for lipogenesis and cholesterogenesis. The greater hepatic availability of propionate with the hay-concentrate diets may have upregulated the mRNA expression of AMP-kinase by triggering gluconeogenesis. Both HQH and concentrate supplementation downregulated genes related to nutrient catabolism, such as fatty acid oxidation or amino acid breakdown. In conclusion, results demonstrate the importance of the type of solid feed for the energetic state and respective signaling in hepatocytes of weaned calves.

## Highlights

• Energy signaling was different in the liver with high-quality hay and hay-concentrate diets.
• High-quality hay induced a higher energetic state and lipogenesis in the liver.
• High-quality hay and hay-concentrate diets decreased fatty acid oxidation in the liver.
• Hay-concentrate diets decreased hepatic breakdown of amino acids.

## Graphical Abstract

**Figure:**
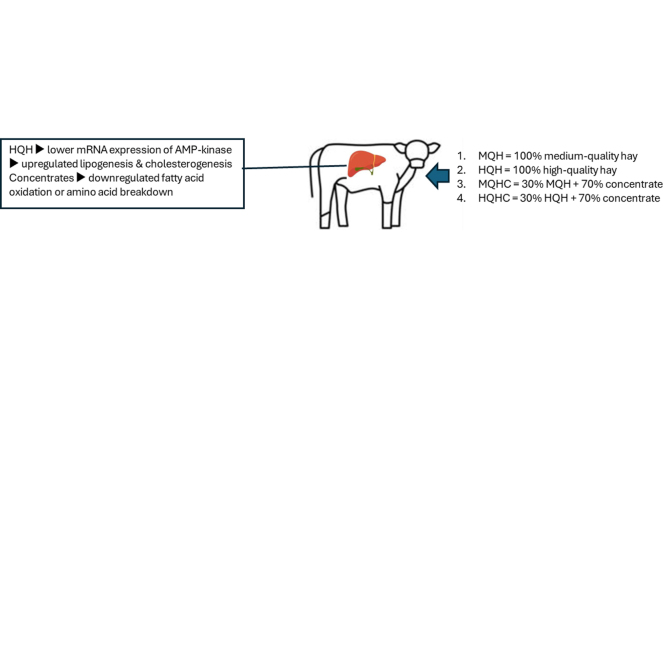
**Summary:** In young calves, the liver plays a central role, especially in priming the energy metabolism and sensing, and this may be affected by early solid feeding. Our study evaluated early solid feeding based on only hay of different qualities or with concentrate supplementation and found that high-quality hay and hay-concentrate diets differently activate energy-sensing transcription factors in the livers of calves. Consequently, downstream signaling regarding anabolic and catabolic pathways diverged between high-quality hay and hay-concentrate diets. High-quality hay stimulated lipogenesis and cholesterogenesis, whereas hay-concentrate diets promoted gluconeogenesis and decreased amino acid breakdown. The present data demonstrate the importance of the type of solid feed for the energetic state and respective signaling in hepatocytes.

The first nutrition of calves is important due to its effects on their development, health, and lifetime performance. The dietary management of calves commonly consists of feeding cereal-rich starters from the first week of life to support rumen development and high growth rates (Yohe et al., 2019). Hay that is harvested at an early maturity stage and dried indoors can support high growth rates in young calves and hence replace starchy concentrates (Terler et al., 2022). This high-quality hay (**HQH**) is rich in water-soluble carbohydrates (**WSC**), CP, and other micronutrients (Klevenhusen and Zebeli, 2021). The differences in carbohydrate profile between concentrates (mainly starch) and HQH (mainly simple sugars and oligosaccharides) result in major differences in the energy substrates generated in the rumen and small intestine (Klevenhusen and Zebeli, 2021) that reach the liver of calves.

Weaned calves, like adult ruminants, predominantly rely on VFA as energy sources. However, their hepatic capacity to utilize these substrates only develops with the onset of solid feed intake. Given the importance of the metabolic role of the liver for optimal adult cow performance (Angeli et al., 2019), it is essential to understand more about the potential impact of feeding regimens on early life hepatic energy metabolism in the early postweaning phase. Therefore, the objective of this study was to determine the effects of feeding different hay qualities, with or without concentrate supplementation, on the mRNA expression of genes related to hepatic lipid and glucose metabolism and cellular energy status in weaned calves. We hypothesized that the HQH, due to its higher WSC content compared with hay with lower protein and higher NDF content, would increase the energetic state of hepatocytes and subsequently upregulate anabolic processes such as lipogenesis. We further hypothesized that the cellular energetic status between calves fed the HQH and calves fed the additional concentrate would be similar, but due to different energetic substrate availability would trigger different anabolic pathways (e.g., lipogenesis).

Liver samples were collected in a previous calf study (Terler et al., 2022; Hartinger et al., 2024). The experimental descriptions, including animal housing, dietary components, feed analyses, and feeding procedures are reported in detail earlier (Terler et al., 2022). In brief, the trial was conducted in a 2 × 2 factorial design with 2 hay qualities and 2 concentrate levels. In total, 40 Holstein calves (17 male and 23 female) were used. The calves were allotted to 4 treatment groups directly after birth: (1) 100% MQH, (2) 30% MQH and 70% concentrate (on fresh matter basis; **MQHC**), (3) 100% HQH, and (4) 30% HQH and 70% concentrate (on fresh matter basis; **HQHC**). Male and female calves were equally distributed among the 4 dietary groups. The chemical composition of the hays and concentrate is provided in Table 1. Calves had access to fresh acidified whole milk (19.2 MJ of ME, 260 g of CP, 322 g of ether extract, and 360 g NFC/kg DM) in a milk feeding bucket with teat, which was freely available in the first 4 wk and afterward gradually reduced until the calves were weaned at the end of wk 12. For acidification, ~0.1% of 85% formic acid was added to raw whole milk to reach a pH of 5.5 (Terler et al., 2022). More details on the feeding protocol can be found in Terler et al. (2022). Calves were kept in adaptable individual boxes on straw, with visual contact with the other calves. The calves had free access to their respective diets throughout the experiment.

**Table 1:** Chemical composition (mean ± SD) of solid feeds fed to experimental Holstein calves (adapted from Terler et al., 2022)

Item^1^ (g/kg DM unless noted)	Medium-quality hay	High-quality hay	Concentrate
DM (g/kg)	899 ± 24	877 ± 30	891 ± 13
CP	149 ± 29	210 ± 11	193 ± 9
Ether extract	18 ± 3	24 ± 3	18 ± 2
Ash	76 ± 7	86 ± 3	39 ± 11
NDF	522 ± 24	455 ± 15	204 ± 12
ADF	329 ± 15	247 ± 11	66 ± 5
ADL	49 ± 7	23 ± 3	13 ± 2
NFC	235 ± 34	225 ± 16	547 ± 16
Water-soluble carbohydrates	124 ± 34	205 ± 10	—
Ethanol-soluble carbohydrates	99 ± 27	167 ± 4	—
Fructans	25 ± 10	38 ± 10	—
ME (MJ/kg DM)	9.4 ± 0.4	11.2 ± 0.2	13.5 ± 0.2
peNDF >8 mm (% DM)	38.0 ± 6.3	43.1 ± 0.5	—

1 peNDF >8 mm = physically effective NDF >8 mm.

Before establishing the experimental replicates of 5 calves per treatment, a priori power analysis was performed using PROC Power in SAS (version 9.4; SAS Institute, Cary, NC) according to Castelloe (2000). For this analysis, we assumed to detect a difference with biological relevance between hay versus concentrate for an effect size *F* of 1.0 to 1.5 and standard variation *s* of ~0.25 to 0.30. A priori information regarding *F* and *s* were taken from previous data for rumen and gut mRNA expression (Hartinger et al., 2024). The power analysis indicated 5 as the minimum number of biological replicates allowing a statistical power (Power 1-β error probability) of ~80% to 85% (varying from 75% to 88% depending on target variables pretested) with α = 0.05 able to reject the null hypothesis *H_0_*, if this was true. Accordingly, 5 calves per treatment group, out of 10 animals, were selected for sampling and slaughtered at an age of 100 ± 4 d (average BW of 128.8 ± 19.8 [SD] kg). Originally, only male calves were to be slaughtered to keep the females as replacement heifers. Due to an opposite-sex twin birth and the exclusion of 1 male calf from the experiment, 3 female calves were included in this investigation. Consequently, the MQH, MQHC, and HQHC groups each comprised 4 male calves and 1 female calf, whereas the HQH group consisted of only male calves. The calves had access to feed until 1700 h the day before slaughter. Water was available until slaughter at 0800 h in the morning. Each calf was stunned with a captive bolt gun and bled by cutting the throat. The abdominal cavity was opened, and the rumen and gastrointestinal tract were removed, after which the liver was taken out. The liver tissue samples were collected from the lobe close to the gallbladder. This sample was first rinsed with ice-cold 1× PBS buffer (pH 7.4) to remove blood, blotted dry on a paper towel, cut into pieces of 2 mm × 2 mm, snap frozen in liquid nitrogen, and placed in 2 mL cryotubes (Sarstedt AG & Co. KG, Nümbrecht, Germany) before being stored at −80°C until RNA extraction.

Total RNA was isolated from 30-mg liver tissue samples using the RNeasy Mini Qiacube Kit (Qiagen, Hilden, Germany) similar to the protocol described in Hartinger et al. (2024). The RNA integrity numbers ranged from 7.9 to 8.5. We amplified 3 reference genes (*OAZ1*, *ACTB*, and *RPL19*) to control for mRNA content and 21 target genes. Detailed information on oligonucleotide sequences of the primers, accession numbers of respective genes, and primer efficiency is available from the corresponding author upon request. The geometric mean of the 3 reference genes was used to normalize the raw gene expression and determine the delta threshold cycle (**Ct**) values. The ΔΔCt values were calculated using the average ΔCt of the target gene for the respective dietary group. The 2^−ΔΔCt^ method was used to calculate the relative gene expression.

Data for the hepatic mRNA expression were analyzed by ANOVA using PROC MIXED in SAS. Fixed effects were hay quality, concentrate supplementation, and their interaction, whereas the animal was considered as a random effect. Degrees of freedom were approximated by the Kenward–Roger method (ddfm = kr). Data were reported as the least squares means ± SEM. Multiple pairwise comparisons among LSM were performed using the pdiff option. A significant difference was defined at *P* ≤ 0.05 and trends at 0.05 < *P* ≤ 0.10. Pearson correlation coefficients among the mRNA expression levels were calculated using PROC CORR in SAS. To visualize the correlations, heatmaps were generated using the Hmisc package (version 5.1-1) in R studio (version 1.4.1106; Posit Software, PBC, Boston, MA, http://www.posit.co/).

The quality of hay influenced the hepatic mRNA expression of fatty acid transporters and binding proteins. Calves fed HQH had, on average, a 37% and 80% higher mRNA expression of *MCT1* (*P* = 0.067) and *FABP1* (*P* < 0.001), respectively, compared with calves fed MQH (Table 2). By contrast, expression of *MCT2* was 26% lower (*P* = 0.037) in hepatocytes of calves fed HQH and HQHC diets compared with those fed the MQH and MQHC diets. Concentrate supplementation tended (*P* = 0.055) to increase the mRNA expression of *MCT4* by 43% compared with only hay feeding. Both the quality of hay and supplementation of concentrate affected the mRNA expression of *AMPK* and *PPARG* in hepatocytes. The HQH decreased the mRNA expression of *AMPK* (*P* = 0.059) and *PPARG* (*P* = 0.013) by 24% and 25%, respectively, compared with MQH, whereas the supplementation of concentrate increased the mRNA expression compared with only hay (*P* < 0.05). Similarly, concentrate addition tended (*P* = 0.086) to increase the mRNA expression of *ADIPOR1* compared with hay-only feeding. The hay × concentrate interactions for the mRNA expression of *ACACA* (*P* = 0.037) and *HMGCR* (*P* = 0.056) indicated that when the concentrate was added to the HQH their mRNA expression was downregulated and similar to the mRNA expression in hepatocytes of calves fed the MQHC. Moreover, the mRNA expression of *HMGCS1* tended (*P* = 0.078) to be 51% higher in hepatocytes from calves fed the HQH compared with those fed the MQH. The mRNA expression of *ACADM* tended (*P* = 0.054) to be 16% lower with HQH compared with MQH, whereas it decreased (*P* = 0.018) by 20% due to the concentrate supplementation. The supplementation of concentrate decreased (*P* = 0.011) the mRNA expression of *MMUT* by 21% compared with the diets without concentrate. Calves fed HQH tended to have a lower mRNA expression of *SLC2A2* but higher expression of *G6PC1* compared with calves fed MQH (*P* < 0.10). Concentrate upregulated (*P* = 0.044) the mRNA expression of *PCK1* by 75% compared with diets without concentrate. Correlation analysis indicated associations between mRNA expression levels of fatty acid transporters, energy-sensing transcription factors, and down-stream genes related to anabolic (lipidogenesis, cholesterogenesis, and ketogenesis) and catabolic metabolism (e.g., β-oxidation and amino acid catabolism; Figure 1).

**Table 2:** Relative mRNA expressions in the liver of calves fed starter diets differing in hay quality (medium and high) and concentrate supplementation (0% and 70%)^1^

Item	Medium		High		SEM	*P*-value		
	0%	70%	0%	70%		H	C	H × C
Energy status mediating transcription factors and receptors								
*AMPK*	1.03	1.63	0.84	1.19	0.157	0.059	0.008	0.448
*PPARG*	1.02	1.28	0.76	0.96	0.102	0.013	0.040	0.754
*PPARA*	1.03	1.55	1.49	2.23	0.487	0.261	0.215	0.818
*ADIPOR1*	1.04	1.58	0.81	1.38	0.304	0.482	0.086	0.961
*ADIPOR2*	1.28	1.89	1.82	0.80	0.404	0.516	0.621	0.061
Fatty acid synthesis and transport								
*ACACA*	1.02	1.47	1.74	1.43	0.167	0.060	0.680	0.037
*FASN*	1.62	0.83	0.64	0.96	0.497	0.407	0.643	0.281
*FABP1*	1.05	1.11	2.23	1.66	0.199	<0.001	0.217	0.133
Cholesterol biosynthesis								
*HMGCR*	1.07	1.88	3.02	1.79	0.496	0.080	0.679	0.056
*HMGCS1*	1.14	1.26	2.29	1.32	0.325	0.078	0.211	0.112
Catabolism of amino acid and odd-chain fatty acids								
*MMUT*	1.00	0.79	0.94	0.79	0.061	0.599	0.011	0.596
Ketone body metabolism								
*BDH2*	1.03	0.98	0.83	1.05	0.141	0.642	0.551	0.366
Fatty acid oxidation in mitochondria								
*ACADM*	1.01	0.79	0.83	0.68	0.070	0.054	0.018	0.636
*ACADS*	1.06	0.95	0.97	0.93	0.177	0.752	0.663	0.859
*PLA2G2D4*	1.38	2.27	1.37	1.52	0.590	0.528	0.395	0.539
Glucose metabolism								
*SLC2A2*	1.01	1.04	0.89	0.84	0.089	0.095	0.886	0.658
*G6PC1*	1.02	1.07	1.19	1.31	0.098	0.053	0.396	0.680
*PCK1*	1.02	1.36	1.16	2.47	0.376	0.115	0.044	0.217

1 Values are LSM ± SEM. H = hay quality; C = concentrate supplementation.

**Figure 1:**
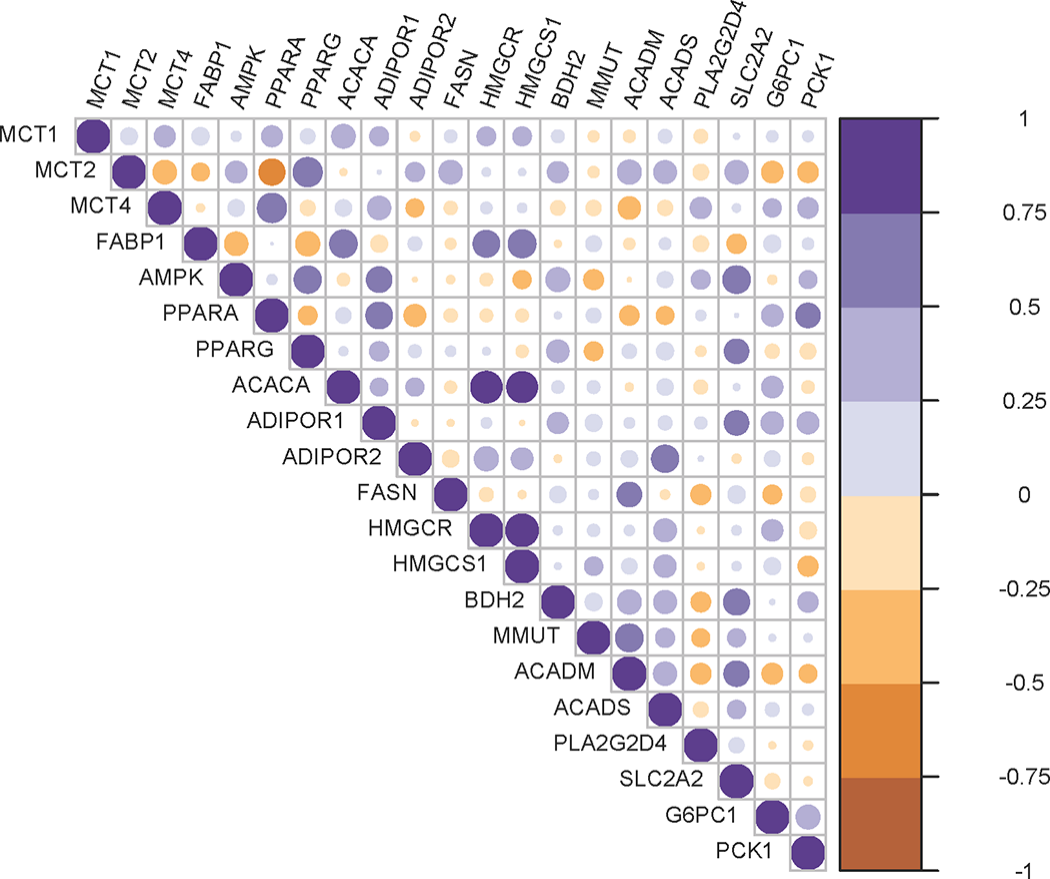
Heatmap illustrating Pearson correlations between mRNA expression levels. Pearson correlations were considered relevant with *P* < 0.05 and r > 0.45 or r < −0.45.

The present research was based on recent findings of our group showing that feeding of HQH and hay-concentrate diets to postweaning calves stimulated the DMI by ~5 kg in wk 13 and 14 of life compared with MQH (Terler et al., 2022), which supported higher weight gain of calves fed the HQH and hay-concentrate diets. Interestingly, despite similar ME intake, the weight gain was 300 g lower in the MQHC group compared with the HQH group in wk 13 and 14 (Terler et al., 2022), indicating that differences in energy metabolism may have caused this discrepancy. This assumption was supported by differences in fermentation products measured in the rumen and gut (Hartinger et al., 2024). Accordingly, the hay-only diets promoted acetate and butyrate fermentation in the rumen compared with hay-concentrate diets, whereby the HQH provided more VFA and more propionate but less acetate from ruminal fermentation compared with the MQH (Hartinger et al., 2024). This resulted in different composition and amounts of glucogenic and lipogenic precursors reaching the liver via the ruminal and portal veins. Moreover, more propionate reached the liver of weaned calves fed the hay-concentrate diets compared with the hay-only diets (Hartinger et al., 2024). To explain the observations related to the weight gain, the current study aimed to explore the changes in hepatic energy metabolism due to the different hay qualities and concentrate supplementation. Differences in hepatic substrate provision between hay qualities were supported by the changes in mRNA expression levels of *MCT1* and *MCT2*. Increased flow of acetate, butyrate, or lactate between the 2 hay qualities may be responsible for the increased *MCT1* expression with HQH. Among the various monocarboxylate transporters (**MCT**), MCT-2 has the highest affinity for pyruvate (Lin et al., 1998), which may have been lower in calves fed the HQH compared with calves fed the MQH. Concentrate supplementation increased *MCT4* mRNA expression compared with hay-only feeding, supporting higher flow of propionate, lactate, or both, to the liver.

Adenosine monophosphate-kinase (**AMPK**) serves as a metabolic sensor in hepatocytes in response to alteration in cellular energy charge and is upregulated when the intracellular AMP/ATP ratio increases (McFadden and Corl, 2009). Accordingly, the ATP levels in hepatocytes of calves fed the MQH were lower compared with those of calves fed the HQH. Consequently, the *AMPK* mRNA expression may have reflected the generated VFA levels in the rumen and gut in the calves fed the respective diets. It seemed that not only did the total amount of VFA play a role, but also that the VFA profile influenced the *AMPK* mRNA expression. Higher acetate and butyrate levels with the HQH seemed to be more effective at decreasing the *AMPK* mRNA expression than propionate with the hay-concentrate diets. We can assume that propionate reaching the liver from the rumen and gut increased gluconeogenesis, which was indicated by the upregulation of the first rate-limiting enzyme in gluconeogenesis (*PCK1*) mRNA expression (Méndez-Lucas et al., 2013) in calves fed the hay-concentrate diets. If the signaling in bovine hepatocytes is similar to observations made for hepatocellular carcinoma cells, expression of *PCK*1 regulates AMPK and cell proliferation (Tuo et al., 2019). This connects gluconeogenesis to AMPK activation and the fasting state. Although glucose supply by gluconeogenesis is the normal condition in adult ruminants, gluconeogenetic pathways may be still related to the fasting condition in calves shortly after weaning. This assumption is supported by the tendency for a higher expression of the adiponectin receptor *ADIPOR1* with the hay-concentrate feeding and positive correlation between mRNA expression levels of *ADIPOR1* and *AMPK*. Fasting increases adiponectin, which in turn stimulates AMPK activity and appetite (Chen et al., 2013). Hence, activation of AMPK signaling may have been one underlying stimulus for the higher DMI but lower weight gain in the calves fed the hay-concentrate diets when compared with the calves fed the MQH and HQH diets, respectively (Terler et al., 2022).

When the cellular AMPK activity rises, it activates catabolic ATP-generating processes to restore intracellular energy (McFadden and Corl, 2009). Consequently, we observed tendencies for higher mRNA expression levels of *ACACA*, *HMGCR*, and *HMGCS1* in calves fed the HQH compared with calves fed the MQH. As a binding and transport protein of long-chain fatty acids, the increased mRNA expression of *FABP1* may support increased fatty acid synthesis in calves fed the HQH compared with those fed the MQH. The fact that the total amount of acetate reaching the liver with the HQH was higher than with the MQH may explain the higher mRNA expression levels of *HMGCR* and *HMGCS1* with the HQH. The supplementation of the concentrate to the HQH diet diminished the beneficial effect of HQH on lipogenesis and cholesterogenesis and was probably related to the higher *AMPK* and *PCK1* mRNA expression.

In addition to AMPK, the nuclear receptor peroxisome-proliferation-activated receptor (PPAR)-α integrates nutritional signals to regulate transcriptional networks for hepatic β-oxidation and ketogenesis (Berthier et al., 2021). However, *PPARA* as well as *BDH2*, which is the enzyme that reduces acetoacetate to BHB, were similarly expressed among diets. Nevertheless, the positive correlation between mRNA expression levels of *AMPK* and *BDH2* supports that a lower cellular ATP level triggers the expression of ketogenic enzymes.

Both the HQH and concentrate supplementation lowered the mRNA expression of *ACADM* compared with the MQH diet, indicating lower medium-chain fatty acid oxidation. The mRNA expression of *ACADS* was not changed by diet, but its positive correlation with the mRNA expression of *ACADM* may indicate a similar trend of a downregulation of mitochondrial β-oxidation. This observation contrasts the findings for *AMPK* mRNA expression for the hay-concentrate diets. According to the signal transduction of *AMPK*, we would have expected a higher expression with the hay-concentrate diets but we found lower mRNA expression of *ACADM* compared with the MQH. This discrepancy indicated different signal transduction pathways with the hay-concentrate diets compared with the HQH diet. The *ACADM* mRNA expression may have been regulated by the nuclear factor PPAR-γ in the calves fed the hay-concentrate diets. Consequently, the metabolic state of the calves fed the hay-concentrate diets may have resembled more the metabolic state of an adult cow. In the fed state, the primary role of PPAR-γ is to increase the uptake of fatty acids and glucose into hepatocytes and stimulate hepatic lipogenesis, simultaneously inhibiting β-oxidation (Berthier et al., 2021). The positive correlation between mRNA expression levels between *SLC2A2* and *PPARG* supports the role of PPAR-γ to stimulate glucose uptake in hepatocytes (Berthier et al., 2021). The mRNA expression levels of *ACADM* positively correlated with those of *MMUT*, supporting the downregulation of catabolic processes (in hepatocytes of these calves compared those fed the hay-only diet, which again may be reflective of a more adult metabolism). The mRNA expression levels of *SLC2A2* and *G6PC1* were differently regulated as response to the HQH compared with the MQH and may correspond to the glucose or propionate concentrations reaching the liver via the portal vein.

Although we only assessed the mRNA expression of a limited number of key enzymes in various pathways related to energy metabolism in weaned calves, the present data demonstrate the importance of the type of solid feed for the energetic state and respective signaling in hepatocytes. The observed mRNA expression levels in calves fed the hay-concentrate diets versus the hay-only diets may be indicative for a transitory phase to reach the adult metabolic phase. Although upregulated AMPK signaling in these calves may have stimulated appetite, the simultaneous stimulation of catabolic processes may have prevented more body weight gain compared with the hay-only diets. Feeding HQH instead of MQH, in turn, led to trends for anabolic signaling in the liver, potentially contributing to the superior weight gain in calves fed this type of hay.
